# Transcriptomic Analysis of the Spider Venom Gland Reveals Venom Diversity and Species Consanguinity

**DOI:** 10.3390/toxins11020068

**Published:** 2019-01-24

**Authors:** Zhaotun Hu, Bo Chen, Zhen Xiao, Xi Zhou, Zhonghua Liu

**Affiliations:** 1 Key Laboratory of Research and Utilization of Ethnomedicinal Plant Resources of Hunan Province, College of Biological and Food Engineering, Huaihua University, Huaihua 418008, China; huzhaotun@hhtc.edu.cn; 2 The National and Local Joint Engineering Laboratory of Animal Peptide Drug Development, College of Life Sciences, Hunan Normal University, Changsha 410081, China; hnnuchenboo@sina.com (B.C.); Zhen-xiao@outlook.com (Z.X.)

**Keywords:** *Selenocosmia jiafu*, spider, venom gland, transcriptomic analysis, cDNA library, diversity

## Abstract

*Selenocosmia jiafu* (*S. jiafu*) has been recently identified as a new species of spider in China. It lives in the same habitat as various other venomous spiders, including *Chilobrachys jingzhao* (*C. jingzhao*), *Selenocosmia huwena* (*S. huwena*), and *Macrothele raveni* (*M. raveni*). The venom from these different species of spiders exhibits some similarities and some differences in terms of their biochemical and electrophysiological properties. With the objective to illustrate the diversity in venom peptide toxins and to establish the evolutionary relationship between different spider species, we first performed transcriptomic analysis on a cDNA library from the venom gland of *S. jiafu*. We identified 146 novel toxin-like sequences, which were classified into eighteen different superfamilies. This transcriptome was then compared with that of *C. jingzhao*, which revealed that the putative toxins from both spider venoms may have originated from the same ancestor, although novel toxins evolved independently in the two species. A BLAST search and pharmacological analysis revealed that the two venoms have similar sodium channel modulation activity. This study provides insights into the venom of two closely related species of spider, which will prove useful towards understanding the structure and function of their toxins.

## 1. Introduction

In the animal kingdom, changes in the living environment drive species evolution enabling survival of the fittest. Spiders are a good example, being one of the most successful venomous animals to inhabit the earth, with more than 150,000 species [1,2,3]. The remarkable evolutionary success of spiders is due in large part to the evolution of a complex venom that ensures rapid capture of prey and defense against predators. Spider peptide toxins have great diversity. Based on a conservative estimate of 200 peptide toxins per venom, there are more than 300 million bioactive peptides in spider venoms globally [4]. Thus, spider venom may provide a good model to study toxin selectivity, structure–activity relationships, and the molecular evolution of peptide toxins [3]. To date, some spider venoms (from *C. jingzhao* and *S. huwena*, for example) have been systematically investigated by high-throughput methods for peptide toxin identification [5,6,7,8].

*S. jiafu* is a venomous species of spider found in the hilly areas of Yunnan and Guangxi province in the south of China [9]. It has the same living habitat as that of *C. jingzhao* and *S. huwena* and their venoms have some similarities in a variety of their toxic components that have varied pharmacological properties. RP-HPLC and MALDI-TOF-MS analysis showed that *S. jiafu* venom contains more than hundreds of peptides with a predominant mass of 3000–4500 Da. Whole-cell patch-clamp analyses indicated that the venom could inhibit voltage-gated sodium channels (including TTX-S and TTX-R), voltage-gated potassium channels, and voltage-gated calcium channels in rat dorsal root ganglion (DRG) neurons [9]. However, the venom components that possess the bioactivity and diversity remain to be explored.

Furthermore, spider venoms are known to contain several classes of peptide toxins that target voltage-gated ion channels and have been considered as a potential source of new compounds with specific pharmacological properties [10,11,12]. The potential of venom components as pharmacological tools and as potential leads for the development of new drugs and pesticides has recently been recognized [12,13]. As a result, venoms have generated broad interest in the scientific community and in the agrochemical and pharmaceutical industries in recent years [11,14]. Venoms from tarantulas are more heterogeneous, and the specific composition of these venoms varies significantly from species to species [6]. The venom from *S. jiafu* could be a novel source for the identification of novel peptide toxins acting on ion channels and receptors.

Due to limited access to the crude venom from *S. jiafu*, it is a daunting task to carry out investigation of individual toxins through venom purification. Venom gland transcriptomic analysis may be employed as a clever alternative to determine the sequences of cDNA that encode specific peptide toxins. In light of that, transcriptomic analysis of the venom gland from *S. jiafu* was conducted in the present study. As a result, 752 high-quality expressed sequence tags (ESTs) were generated and 146 novel putative toxin sequences were identified. When compared with that of *C. jingzhao*, our data revealed that the putative toxins from both spiders may have originated from the same gene ancestor.

## 2. Results and Discussion

### 2.1. cDNA Library and EST Analysis

**Figure 1:**
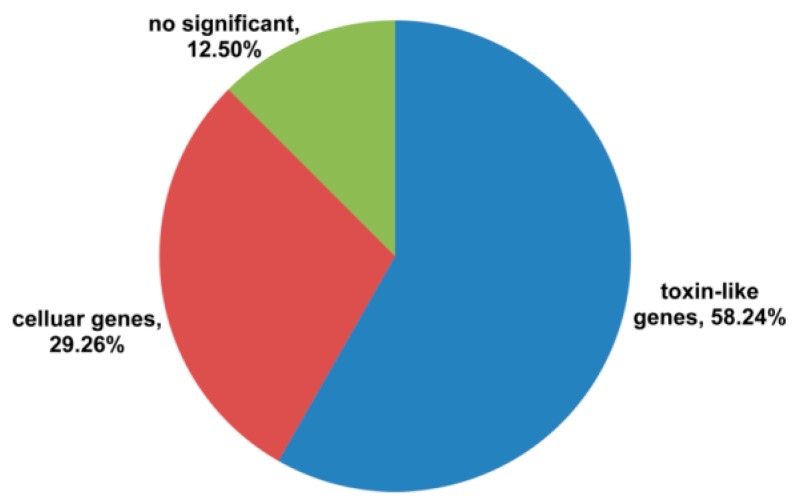
Relative proportion of each transcript category from *S. jiafu* venom gland cDNA library.

The directional full-length cDNA library was generated from the venom glands of *S. jiafu*. The average length of cDNA in the library was about 750 bp, ranging from 0.3 to 3.0 kb. Clones (1299) were randomly sequenced to generate 752 high-quality ESTs from the cDNA library. Of the 752 ESTs, (1) 58.24% (438 of 752 ESTs) encoded putative toxin precursors, and of these, 257 non-redundant sequences encoded 146 novel putative toxin precursors; (2) 29.26% (220 of 752 ESTs) were similar to cellular transcripts, and 12.5% (94 of 752 ESTs) had no significant similarity to any known sequences, as shown in Figure 1. From the 146 putative toxin precursors, 99 non-redundant mature peptide sequences were obtained.

**Figure 2:**
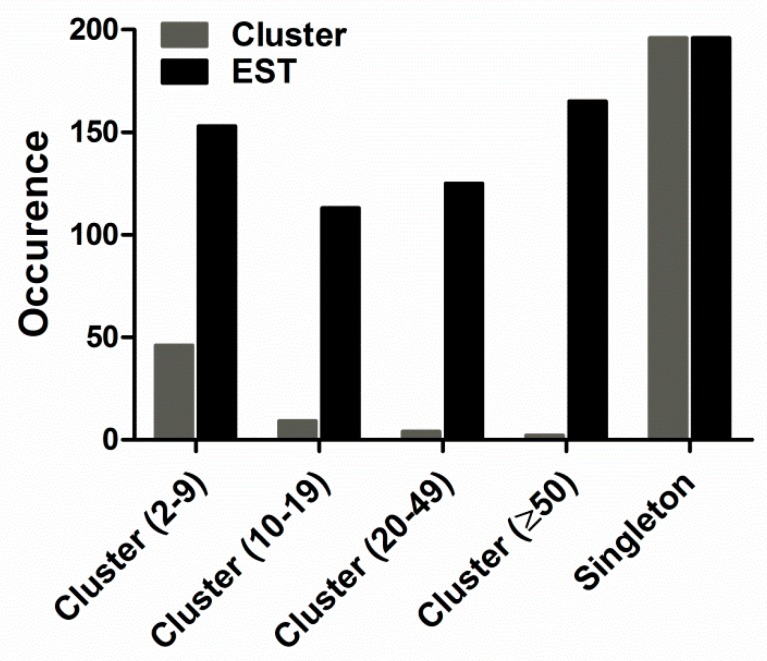
Prevalence distribution of the cluster size. The initial 752 expressed sequence tags (ESTs) were grouped into 61 contigs and 196 singletons.

Additionally, the 752 assembled ESTs resulted in 257 clusters, including 61 contigs and 196 singletons. The abundance distribution of all ESTs was cataloged as shown in Figure 2: (1) Two clusters containing more than 50 ESTs each, represented the most abundant transcripts. They constituted 0.78% of the total clusters (2 of 257 clusters) and 21.94% of the total ESTs (165 of 752 ESTs). All of them were predicted to encode toxin proteins. (2) Four clusters containing 20–49 ESTs each, represented 1.56% of the total clusters (4 of 257 clusters) and 16.62% of the total ESTs (125 of 752 ESTs). All of them were predicted to encode toxin proteins. (3) Nine clusters containing 10–19 ESTs each, represented 3.50% of the total clusters (9 of 257 clusters) and 15.03% of the total ESTs (113 of 752 ESTs). Of the nine clusters, seven encoded toxin proteins and two encoded cellular body proteins. (4) The 46 low-abundance clusters, each with 2-9 ESTs, constituted 20.35% of ESTs (153 of 752 ESTs) and 17.90% of the total clusters (46 of 257 clusters). Of the 46 clusters, 11 encoded toxin proteins and 35 encoded cellular body proteins. (5) 196 singletons representing 26.06% of ESTs (196 of 752 ESTs) and 76.26% of the total clusters (196 of 257 clusters), were unique ESTs and their occurrence rate was only once in the library.

### 2.2. Classification of Toxin-Like Precursors

**Figure 3:**
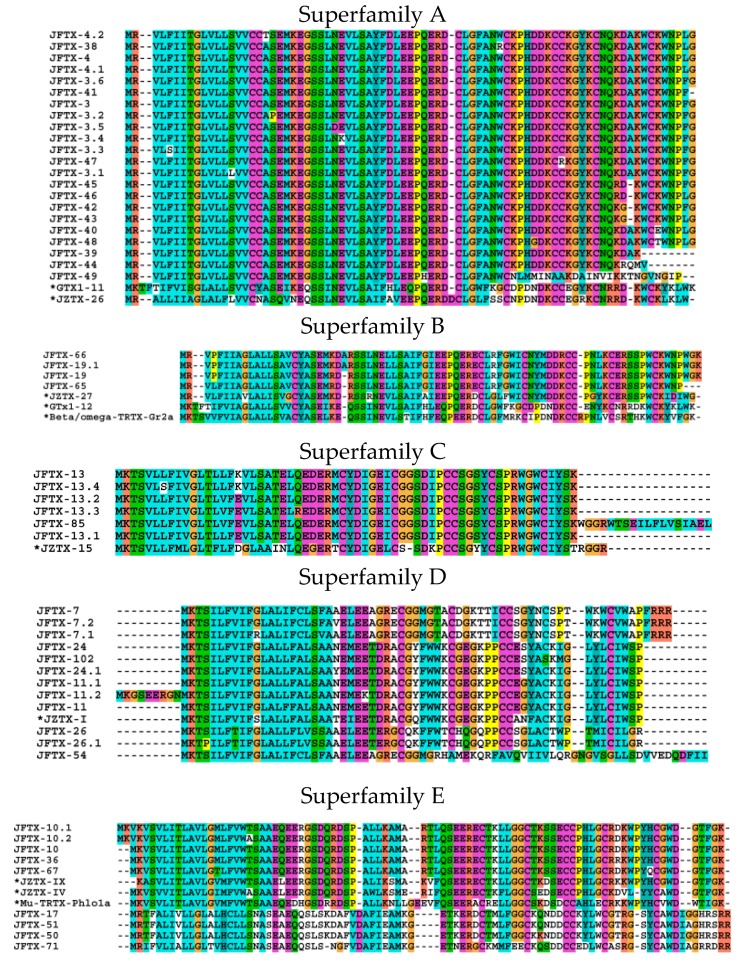

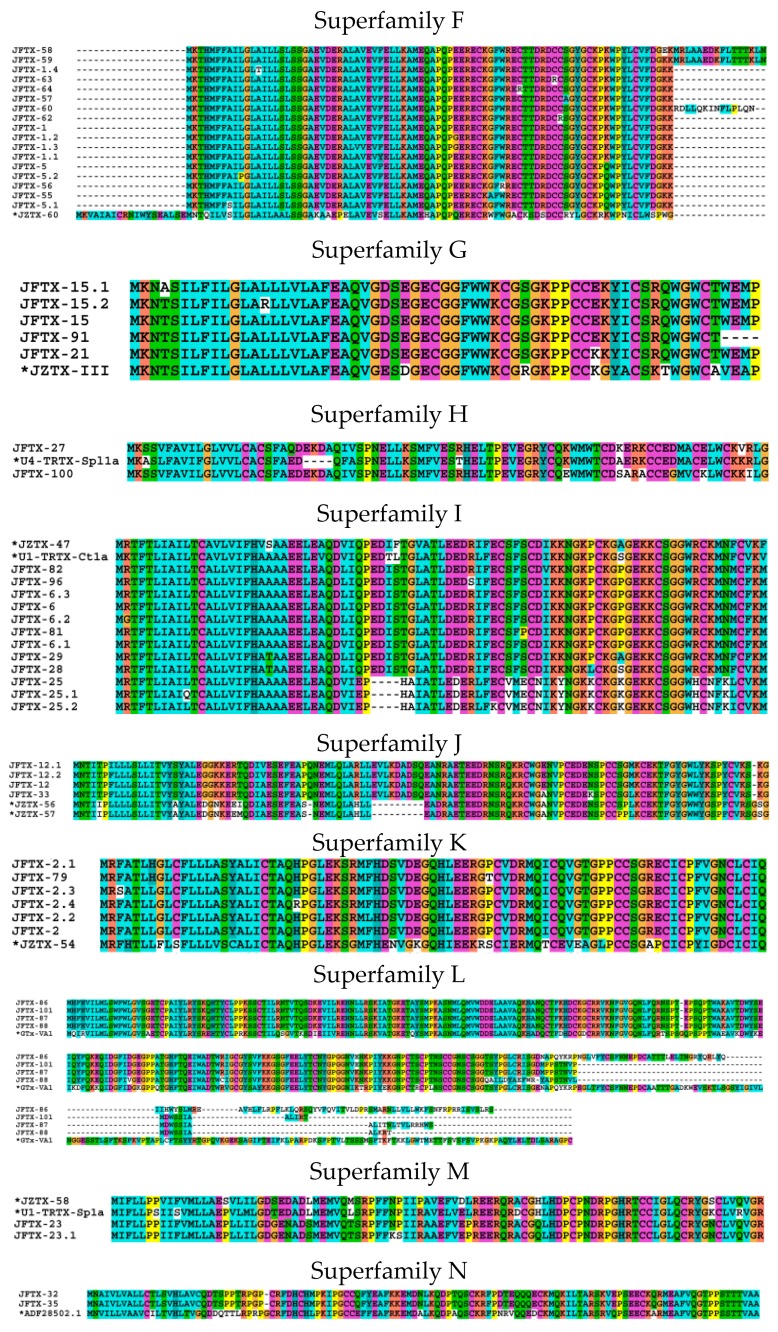

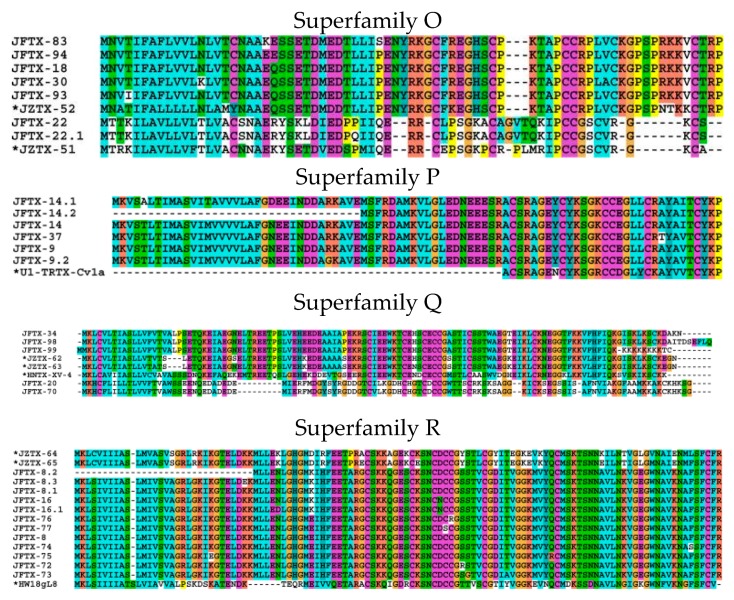
Multiple sequence alignment of putative toxin precursors from the cDNA library of *S. jiafu* (Superfamily **A**–**R**). Toxins from other spiders are marked with asterisk dots.

All the putative toxin precursors from this cDNA library were classified into 18 superfamilies (A-R) according to their cysteine pattern and phylogenetic analysis, as shown in Figure 3 and Figure S1. Any sequence containing two or more cysteine residues and a signal peptide was considered to be a toxin sequence. Based on these criteria, 438 toxin peptides and 146 full-length toxin precursors were obtained from the cDNA library (including precursor peptides, signal peptides, and mature peptides). Of the 146 toxin precursors, 99 non-redundant mature peptides were obtained, because some toxin precursors have the different precursor peptides and signal peptides. Of which, 48 mature peptides were screened against online software (http://web.expasy.org/blast/) to obtain sequence similarity with toxins. A BLAST search showed that these putative toxins shared high similarity with *C. jingzhao*. Through MEGA 7 software, the phylogenetic tree of 146 toxin precursors was drawn using the neighbor-joining method. The results indicated that these toxin precursors could be classified into 18 families (A-R) according to phylogenetic analysis and cysteine patterns, as shown in Figure 3 and Figure S1.

#### 2.2.1. Superfamily A

The superfamily A was the most abundant cluster in this library, comprising of 22 putative toxin precursors. This superfamily showed a high sequence similarity, except when several sequences had a residue mutation. Additionally, the precursor peptides had a “PQER” sequence, which is the cleavage site of the propeptide [15,16]. Some of the precursors contained a single residue “G” at the C-terminal, indicating C-terminal amidation during post-translational processing. Furthermore, except for JFTX39, JFTX44, JFTX47, and JFTX49, all other mature peptides contained six cysteine residues and the same cysteine pattern (“*X*_1_C*X*_6_C*X*_6_CC*X*_4_C*X*_6-7_C*X*_5_”; *X* is any amino acid), which was extremely common in other identified spider toxins [14]. This spatial structure is likely to be the inhibitor cysteine knot (ICK) motif, and these sequences share high similarity with GTX1-11 (69%) and JZTX-26 (65%). GTX1-11 is a 35-residue long toxin molecule from the venison glands of *Grammostola rosea* (*G. rosea*). GTX1-11 belongs to the GTX1 family that has an inhibitory effect on sodium channels [17,18]. The function of JZTX-26 remains unknown.

#### 2.2.2. Superfamily B

The superfamily B includes four similar sequences, which contain the same cleavage site (PQER sequence), and the cysteine framework of the predicted mature toxin—*X*_1_C*X*_6_C*X*_6_CC*X*_4_C*X*_6_C*X_n _* (X is any amino acid, and n is an uncertain number). This family shows a high sequence identity (77%) with the toxin JZTX-27 from *C. jingzhao*. In our previous report, JZTX-27 was described to be a potent gating modifier toxin that inhibited bacterial sodium channels and regulated mammalian sodium channels [19]. Interestingly, the putative toxin precursors of family B have relatively high sequence identity (58%) with beta/omega-TRTX-Gr2a (GpTx-1) from the spider *G. rosea* that is a potent and selective Na_V_1.7 antagonist [17]. Based on these, we predict that it may be a gating modifier which affects sodium channel currents.

#### 2.2.3. Superfamily C

The superfamily C includes the JFTX-13 and JFTX-85 families that have the conserved signal peptides and propeptide regions, and the same cleavage site (EDER sequence). This superfamily has the same cysteine framework of the predicted mature toxin from superfamily B. JFTX-13 shows a high sequence identity (75%) with JZTX-15 from *C. jingzhao* [8].

#### 2.2.4. Superfamily D

The superfamily D has twelve homologous members that are clustered into six toxins (JFTX-7, JFTX-11, JFTX-24, JFTX-26, JFTX-54, and JFTX-102). Except for JFTX-102 which has a single site mutation (C/S) and JFTX-54 that has only cysteine, all other toxins contained six residues and formed the cysteine pattern *X*_1_C*X*_6_C*X*_6_CC*X*_4_C*X*_6_C*X_n_*. JFTX-24 shares 89% identity with JZTX-I from the spider *C. jingzhao*. JZTX-I has been shown by our group to be a gating modifier that inhibited sodium channel fast inactivation [20,21]. This indicates that superfamily D toxins might possess the potential for sodium channel activity.

#### 2.2.5. Superfamily E

The superfamily E contains nine transcripts clustered into seven toxins. The signal peptide sequence of the putative toxin precursors in this family has a high similarity, the propeptide consists of 24–29 amino acid residues and the cleavage sites are “SEER” or “TKER”. The mature peptide contains six cysteine residues forming the cysteine pattern *X*_1_C*X*_6_C*X*_5_CC*X*_4_C*X*_6/7_C*X_n_*, and the C-terminus had the amidation signal sequences “GK” or “RR”. Sequence analysis showed that JFTX-10 is similar to JZTX-IX [22] (identity 87%) and JZTX-IV [23] (identity 72%) both from *C. jingzhao* and also to Mu-TRTX-Phlo1a (identity 72%) from *Phlogius sp.* tarantula [24]. All three toxins show sodium channel inhibitory activity. JFTX-17 presents 61% similarity with Hanatoxin-2 (HaTx2), a 35-amino acid peptide isolated from the venom of *G. rosea*. HaTx2 is an inhibitor of voltage-gated Kv2.1 potassium channel [25]. The sequence similarity analysis indicated that superfamily E toxins might possess the potential for sodium channel and potassium channel inhibition.

#### 2.2.6. Superfamily F

There are 17 putative toxin precursors in this superfamily. They share over 90% sequence identity. Presumably they may be derived from the same gene ancestor. Additionally, these toxins show high similarity with JZTX-60 (60% identity) from *C. jingzhao*. Like the superfamilies A–E, the mature peptide contains six cysteines that form the cysteine pattern *X*_1_C*X*_6_C*X*_5_CC*X*_4_C*X*_7_C*X_n_*.

#### 2.2.7. Superfamily G

The superfamily G includes the JFTX-15 family, JFTX-21 and JFTX-91. These toxins share a similar sequence, and their precursor peptides do not have a typical propeptide cleavage site. JFTX-21 shares 84% identify with JZTX-III from *C. jingzhao*. JZTX-III contains 36 amino acids that specifically inhibit cardiac sodium channel Nav1.5 and potassium channel Kv2.1 [26,27].

#### 2.2.8. Superfamily H

The superfamily H has two putative toxin precursors (JFTX-27 and JFTX-100); both their propeptides consist of 32 amino acid residues; the cleavage site is “VEGR”; their mature peptides include 30 amino acid residues. JFTX-27 showed 85% sequence identity to U4-TRTX-Spl1a from the venom of the Australian tarantula *Selenotypus plumipes* (*S. plumipes*). U4-TRTX-Spl1a shows insecticidal activity when injected into mealworms [28], making it a possible ion channel inhibitor.

#### 2.2.9. Superfamily I

There are 12 putative toxin precursors in this superfamily. The predicted mature sequences are composed of 39 amino acid residues. Although these mature peptides contain six cysteines, their cysteine pattern (*X*_3_C*X*_3_C*X*_8_C*X*_7_C*X*_5_C*X*_4_C*X*_3_) does not conform to the inhibitor cysteine knot (ICK) motif commonly found in spider toxins, making it different from superfamilies A-H. The mature peptide of JFTX-96 is 74% identical to ω-TRTX-Ba1b from the theraphosid spider *Brachypelma albiceps*. ω-TRTX-Ba1b is an insecticidal peptide lethal for crickets (LD_50_ = 9.2 μg/g). The three-dimensional structure of ω-TRTX-Ba1b revealed a non-ICK fold with a disulfide connectivity of C1–C3, C2–C5, C4–C6 [29]. This pattern differs from the classic ICK motif of spider peptide toxins (C1–C4, C2–C5, C3–C6), such as HNTX-III [30]. JFTX-96 is also similar to JZTX-47 (86% identity) from *C. jingzhao* and U1-TRTX-Ct1a (86% identity). U1-TRTX-Ct1a was isolated from the venom of Australian theraphosid *Coremiocnemis tropix* (*C. tropix*), which contains 39 amino acid residues including six cysteine residues that form three disulfide bonds. Functional analyses showed that Ct1a had no effect on voltage-gated sodium channels from the American cockroach *Periplaneta americana* or the German cockroach *Blattella germanica*, but it was lethal when injected into sheep blowfly *Lucilia cuprina* (LD_50_ = 1687 pmol/g) [2]. Therefore, these data suggest that the superfamily H toxins might possess insecticidal activities similar to ω-TRTX-Ba1b and U1-TRTX-Ct1a. This may explain why injection of the *S. jiafu* venom into the cockroach elicits death [9].

#### 2.2.10. Superfamily J

The superfamily J contains four novel transcripts, including JFTX-12 and its variants. The predicted mature sequences contain cysteines that form a common cysteine pattern *X*_6_C*X*_6_C*X*_6_CC*X*_4_C*X*_14_C*X_n_*. JFTX-12 were similar to JZTX-56 (77% identity) and JZTX-57 (76% identity) both from *C. jingzhao*.

#### 2.2.11. Superfamily K

The superfamily K contains six transcripts that can be clustered into two toxins JFTX-2 and JFTX-79. JFTX-79 is a mutant of JFTX-2, with only one amino acid difference (P/T). Eight cysteine residues formed the pattern *X*_2_C*X*_6_C*X*_7_CC*X*_4_C*X*_2_C*X*_5_C*X*_1_C*X*_2_. JFTX-2 showed a high sequence identity (67%) with the toxin JZTX-54 from the spider *C. jingzhao*.

#### 2.2.12. Superfamily L

Superfamily L includes JFTX-86, JFTX-87, JFTX-88, and JFTX-101. The four putative toxin precursors are identical to a signal peptide sequence with no propeptides. JFTX-86 is composed of 336 amino acid residues and contains 16 cysteine residues, while the remaining members have 14 cysteine residues, with an identical arrangement motif. Sequence BLAST showed that JFTX-86 had 81% identity to GTx-VA1. GTx-VA1 is from the venom gland transcriptome of *G. rosea*, with an as yet unidentified function [18].

#### 2.2.13. Superfamily M

The precursor peptide of the superfamily M includes the single mature peptide JFTX-23, which contains six cysteine residues that form the pattern *X*_3_C*X*_6_C*X*_9_CC*X*_4_C*X*_4_C*X*_4_, and an amidation signal sequence “GR” at the C-terminus. JFTX-23 showed 85% and 83% sequence identities to JZTX—58 from *C. jingzhao* and U1-TRTX-Sp1a from *Selenotypus plumipes*, respectively. JZTX-58′s functional activity is unknown. Although spider toxins have a stable ICK motif to maintain structural stability and reduce protease degradation, their activity is still weakened when taken orally. Interestingly, Hardy et al. reported that U1-TRTX-Sp1a is an orally active insecticidal toxin, also named OAIP-I. The oral LD_50_ for OAIP-1 in the cotton bollworm *Helicoverpa armigera* was 104.26 pmol/g [31]. This implies that JFTX-23 may also be an orally active insecticidal toxin.

#### 2.2.14. Superfamily N

The precursor peptide of the superfamily N includes the signal peptide and the mature peptide, but not the propeptide region, which is different from the known toxins in these venoms. The signal peptides are identical, and their cleavage site is at the second cysteine. The mature peptide contains seven cysteine residues that form the pattern *X*_11_C*X*_4_C*X*_7_CC*X*_20_C*X*_11_C*X*_17_C*X*_20_. The odd number of cysteine residues results in the inability of at least a pair of intrachain disulfide bonds to be formed. Indeed, there are many peptide toxins that contain an odd number of cysteine residues in the spider venom gland, but how disulfide bonds pair still remains unclear. We speculated that interchain disulfide bonds likely assist in the formation of dimers. This approach may increase the diversity of toxins in the venom gland.

#### 2.2.15. Superfamily O

Seven putative toxin precursors belonged to this superfamily. The mature peptide includes six cysteine residues to form the classical ICK motif (-C-C-CC-C-C-). This superfamily of peptides has high identity with JZTX from the *C. jingzhao* venom gland. JFTX-83 showed 80% identity with JZTX-52, and JFTX-22 showed 72% identity with JZTX-51.

#### 2.2.16. Superfamily P

There were six putative toxin precursors in this superfamily. The mature peptides contain six cysteine residues that form the cysteine pattern X_1_CX_6_CX_5_CCX_4_CX_6_CX_3_ and share over 97% sequence homology. A BLAST search of the protein sequence database showed that the mature sequence of JFTX-9 shared 81% identity with U1-TRTX-Cv1a. U1-TRTX-Cv1a is an insect-specific neurotoxic peptide from the venom of *Coremiocnemis validus*, which induced insect-specific non-lethal excitatory activity when injected into crickets, but not in cockroaches and mice [32]. However, its target is as yet unidentified.

#### 2.2.17. Superfamily Q

The superfamily Q contained five putative toxin precursors whose mature peptides contain eight cysteine residues that form a new cysteine pattern (-C-C-C-CC-C-C-C-). They are similar to JZTX-62 (87%) and JZTX-63 (86%) from *C. jingzhao* and HNTX-XV-4 from *Haplopelma hainanum*.

#### 2.2.18. Superfamily R

There were 12 putative toxin precursors in this superfamily. The mature sequences had 65 amino acid residues and the primary structure was similar to JZTX-64 (73%) and JZTX-65 (71%) from *C. jingzhao* and HW18gL8 (55%) from *S. huwena*. Similar to superfamily F, they contain eight cysteine residues in the pattern of “*X*_1_C*X*_7_C*X*_3_C*X*_1_CC*X*_5_C*X*_12_C*X*_26_C*X*_2_”. It was noted that there was a single site mutation (C/R) in JFTX-76 and (C/S) in JFTX-77. This mutation causes the components (-CC-) that form the classical ICK motif to be destroyed, and leads to an odd number of cysteine residues, which results in at least a pair of intrachain disulfide bonds not to be formed.

### 2.3. Phylogenetic Analysis of Putative Toxins from Spiders S. jiafu and C. jingzhao

**Figure 4:**
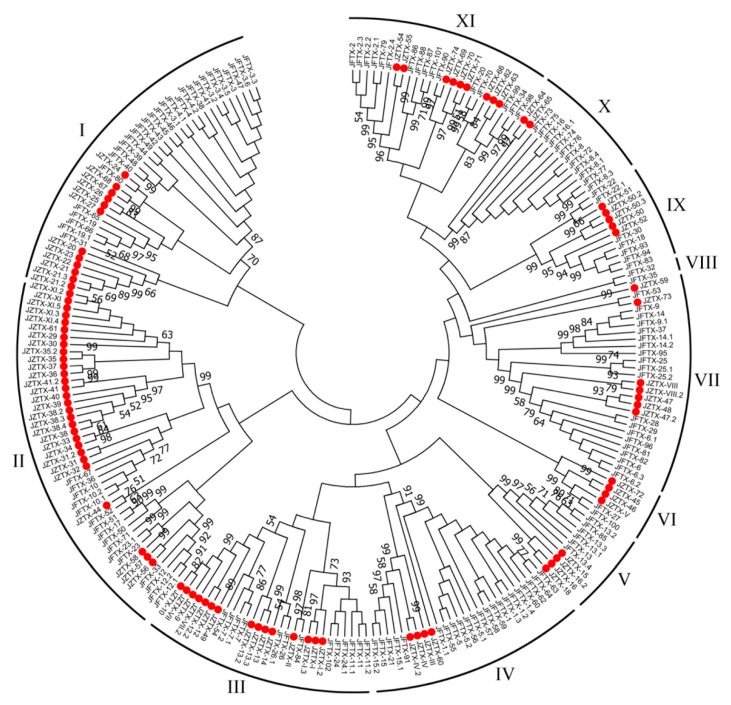
Phylogenetic tree of putative toxin precursors from *S. jiafu* and *C. jingzhao* venom glands. The phylogenetic analysis was conducted using the neighbor-joining method of the MEGA 7 software package. Solid red represents putative toxin precursors from *C. jingzhao*.

As mentioned previously, most putative toxin precursors from *S. jiafu* share very high sequence similarity with those from *C. jingzhao*. Therefore, all putative toxin precursors from the two spiders (145 from the former and 90 from the latter) were mixed and subjected to multiple sequence alignment and phylogenetic analyses. The multiple sequence alignment was performed using ClustalX2, categorizing all 235 precursors into eleven families (I-XI). The phylogenetic tree of all precursors was drawn using the neighbor-joining method. It should be noted that using the two methods generated similar phylogenetic trees. As shown in Figure 4, except for family VIII, all other families contained precursors derived from the two spiders, consistent with the multiple sequence alignment data, showing these precursors to be highly homologous. However, putative toxin precursors from *S. jiafu* share little sequence similarity with those from *S. huwena* and *M. raveni*, two spiders that live in the same habitat with *S. jiafu* and *C. jingzhao*. Bioactivity assays showed that *S. huwena* and *M. raveni* venoms are more lethal to mice after intraperitoneal injections than *S. jiafu and C. jingzhao,* but the activity of *S. huwena* on insects is weaker than *S. jiafu* and *C. jingzhao* [9,33]. These data suggest that peptide toxins from the *S. jiafu* and *C. jingzhao* may have originated from the same gene ancestors, and that the two species shared a close evolutionary relationship.

### 2.4. Pharmacological Activity Analysis of Peptide Toxins from S. jiafu

**Figure 5:**
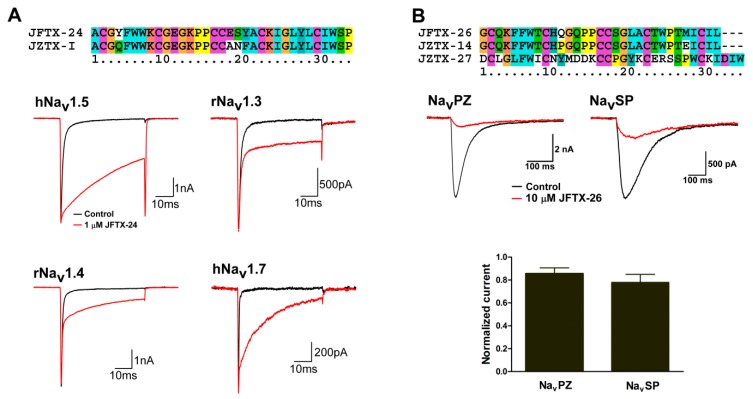
Effect of JFTX-24 and JFTX-26 on voltage gated sodium channels. (**A**) Sequence alignment of JFTX-24 and JZTX-I; 1 μM JFTX-24 inhibits the fast inactivation of hNav1.5, rNav1.3, rNav1.4, and hNav1.7 channels (*n* = 3–5); the currents were elicited by a 50 ms depolarization to –10 mV from a holding potential of –90 mV. (**B**) Sequence alignment of JFTX-26, JZTX-14, and JZTX-27; representative current traces and the bar show that 10 μM JFTX-26 blocks the currents of NavPZ and NavSP channels (*n* = 4); the currents were elicited by a 500 ms depolarization to −20 mV from a holding potential of -100 mV.

In our previous research, we conducted a biochemical and electrophysiological investigation of the crude venom of *S. jiafu*. Whole-cell patch-clamp recording indicated that the venom could inhibit voltage-gated Na^+^, K^+^, and Ca^2+^ channels in rat DRG neurons [9]. The data showed that the venom contains diverse peptides and possesses inhibitory activities on voltage-gated ion channels. However, the characterization of a single toxin had not been reported. Toxins from *C. jingzhao* have been extensively studied. Some peptide toxins have been isolated and analyzed to have a high affinity for voltage-gated ion channels, such as sodium channel gating modifiers JZTX-I, JZTX-III, JZTX-IV, JZTX-27, and JZTX-9 [19,20,21,22,23,26,27]. To further explore the functional activity of *S. jiafu* toxins, we performed RP-HPLC to isolate the peptide toxins from *S. jiafu* venom. We selected two high purity peptide toxins (JFTX-24 and JFTX-26) to test their ion channel activities. The molecular weight was determined by matrix-assisted laser desorption/ionization-time of-flight mass spectroscopy (MALDI–TOF MS). Edman degradation sequencing indicated that JFTX-24 is a 33-residue peptide toxin with three disulfide bonds and shares high identity to JZTX-I (87.9%). JFTX-24 showed similar pharmacological activity to JZTX-I, which significantly inhibited the fast inactivation of the Nav1.5 channel, and mildly affected other sodium channel subunits, including Nav1.3, Nav1.4, and Nav1.7, as shown in Figure 5A. Additionally, we characterized JFTX-26 as an antagonist of the bacterial sodium channel. In this study, 10 μM JFTX-26 inhibited NavPZ and NavSP by 85 ± 5% and 78 ± 7%, as shown in Figure 5B, respectively. As shown in Figure 5B, sequence alignment shows a low identity to JZTX-27 (29%), which was the first reported peptide antagonist for prokaryotic sodium channels. This data suggested that JFTX-26 might be a novel toxin interacting with the bacterial sodium channel. Furthermore, as JFTX-26 shares high identity with JZTX-14 (93%), we speculated that JZTX-14 may possess bacterial sodium channel activity. Consequently, these data suggested that some of the toxins from two spiders with similar sequence patterns generally exhibited similar functions, supporting the idea of them sharing a close evolutionary relationship.

## 3. Conclusions

In summary, a total of 752 high-quality ESTs were identified from the *S. jiafu* venom gland, of which 146 were novel toxin-like sequences. A BLAST search showed that most putative toxin precursors shared a very high sequence similarity with precursors from *C. jingzhao*. A comparative analysis of the two spider venom gland transcriptomes indicated that their toxins might be derived from common gene ancestors, but that some novel toxins could have evolved independently over the course of time. These toxins exhibited structural and functional diversity, and some showed similar pharmacological activity. The venom of *S. jiafu* could be a novel source for the identification of new peptide toxins that act on ion channels and receptors. In our future studies, de novo sequencing of HPLC fractions by mass spectrometry will be used to determine more peptide sequences based on our transcriptomic data. Therefore, the sequence determination and functional prediction of these putative toxins may provide clues for future studies.

## 4. Materials and Methods

### 4.1. cDNA Library Construction

cDNA library construction was as described in our previous study [5,34]. The spiders *S. jiafu* were collected from the hilly area of Ninming county in the Guangxi province. Total RNA was isolated from 12 venom glands of 6 individual spiders using TRIzol Reagent (Invitrogen Corp., Carlsbad, CA, USA). In this study, 1.0 μg total RNA was used for library construction. Full-length cDNA libraries were performed using the Creator^TM^ SMART^TM^ cDNA Library Construction Kit (Clontech Laboratories, Inc, Mountain View, CA, USA), according to the manufacturer’s instructions. cDNA inserts from the individual colonies were amplified by PCR using general M13 forward and reverse primers. The PCR products were analyzed using 1% agarose gel electrophoresis. The cloned inserts were sequenced using an ABI 3730 automatic DNA sequencer according to the manufacturer’s instructions (Shanghai Sangon Biological Engineering Technology and Service Co., Ltd., Shanghai, China).

### 4.2. DNA Sequencing and Bioinformatic Analysis

As described in our previous study [5,34], after removing the Poly-A tail, high-quality sequences were assembled into clusters and short sequences were discarded using SeqMan Pro module of DNASTAR Lasergene software suite. cDNA sequences (contigs and singletons) were used to search against public databases (nr/NCBI, Swiss-Prot +TREMBL/EMBL) using the BlastX program with the e-value cutoff set to <10^−5^ to identify similar sequences and putative functions of the new ESTs [35,36]. Signal peptides were predicted with the SignalP 3.0 program (http//www.cbs.dtu.dk/services/SignalP/) [37]. Multiple sequence alignment was performed using the ClustalX2 program to search for amino acid sequence similarity [38]. The phylogenetic analysis of putative toxins was conducted by MEGA 7 software using the neighbor-joining method [39,40].

### 4.3. Cell Culture and Transfection

HEK293T cells were grown under standard cell culture conditions (5% CO_2_ and 37 °C) in Dulbecco’s modified Eagle’s medium (DMEM, Gibco, Grand Island. NY, USA) supplemented with 10% fetal bovine serum, 2 mM L-glutamine, 100 U/mL penicillin, and 100 µg/mL streptomycin. The cDNA clones of bacterial NavPZ and NavSP were from Professor David E Clapham lab (Janelia Research Campus, Howard Hughes Medical Institute, Ashburn, VA, USA). The mammalian sodium channel cDNA clones (Nav1.3–Nav1.5, Nav1.7) were from Professor Theodore Cummins lab (Stark Neurosciences Research Institute, Indiana University School of Medicine, Indianapolis, IN, USA). Cells were 80–90% confluent before transfection, wild-type rNav1.3, rNav1.4, hNav1.5, NavSP, and NavPZ were transiently transfected into HEK293T cells together with eGFP using Lipofectamine 2000 (Invitrogen, Carlsbad, CA, USA) according to the manufacturer’s instructions. Additionally, hNav1.7 was co-transfected with β1 subunit and β2 subunit. Transfected cells were maintained for 24 h at 37 °C with 5% CO_2_ in electrophysiology experiments. GFP-fluorescence cells were selected for patch-clamp analysis.

### 4.4. Electrophysiology

Whole-cell voltage-clamp recordings were performed using an EPC-10 USB patch-clamp amplifier (HEKA Elektronik, Lambrecht, Germany). Bath solution contained (in mM) 150 NaCl, 2 KCl, 1.5 CaCl_2_, 1 MgCl_2_, 10 HEPES (pH 7.4 with NaOH); the pipette solution contained (in mM) 35 NaCl, 105 CsF, 10 EGTA, 10 HEPES (pH 7.3 with CsOH). The osmolarity of all solutions was maintained at 300–320 mOsm using sucrose. Fire-polished electrodes (2.0–2.5 MΩ) were fabricated from 1.5-mm capillary glass using a P-97 puller (Sutter, Novato, CA, USA). Capacity transients were cancelled, voltage errors were minimized with 80% series resistance compensation. The liquid junction potential between the pipette and bath solutions was zeroed before seal formation. Voltage dependent currents were acquired with Patchmaster at 5 min after establishing whole-cell configuration, sampled at 30 kHz, and filtered at 2.9 kHz. All experiments were conducted at room temperature (25 ± 2 °C).

### 4.5. Study Approval

All of the animal experiments were used according to the guidelines of the National Institutes of Health for the Care and Use of Laboratory Animals. The experiments were approved by the Animal Care and Use Committee of the College of Medicine, Hunan Normal University (identification code: 2018045; date of approval: 9 March 2018).

### 4.6. Data Analysis

Data was analyzed using PatchMaster (HEKA Elektronik, Lambrecht, Germany, 2013) and Igor Pro (WaveMetrics, Lake Oswego, OR, USA) software. All graphs were created using Graphpad Prism 5.01 (GraphPad Software, Inc., La Jolla, CA, USA, 2007). Data was presented as mean ± SEM; *n* represented the number of experimental cells.

## Supplementary Materials

The following are available online at http://www.mdpi.com/2072-6651/11/2/68/s1, Figure S1: Phylogenetic tree of putative toxin precursors from S. jiafu venom glands.
