# Supplementary material for: Transcriptomic Analysis of the Spider Venom Gland Reveals Venom Diversity and Species Consanguinity
Source: Toxins (Basel). 2019 Jan 24;11(2):68. doi: 10.3390/toxins11020068 (PMC6409621; doi:10.3390/toxins11020068)
Supplement: Supplementary file 1 [file toxins-11-00068-s001.pdf]

Supplementary Materials: Transcriptomic Analysis of the Spider Venom Gland Reveals Venom Diversity and Species Consanguinity

Zhaotun Hu, Bo Chen, Zhen Xiao, Xi Zhou and Zhonghua Liu

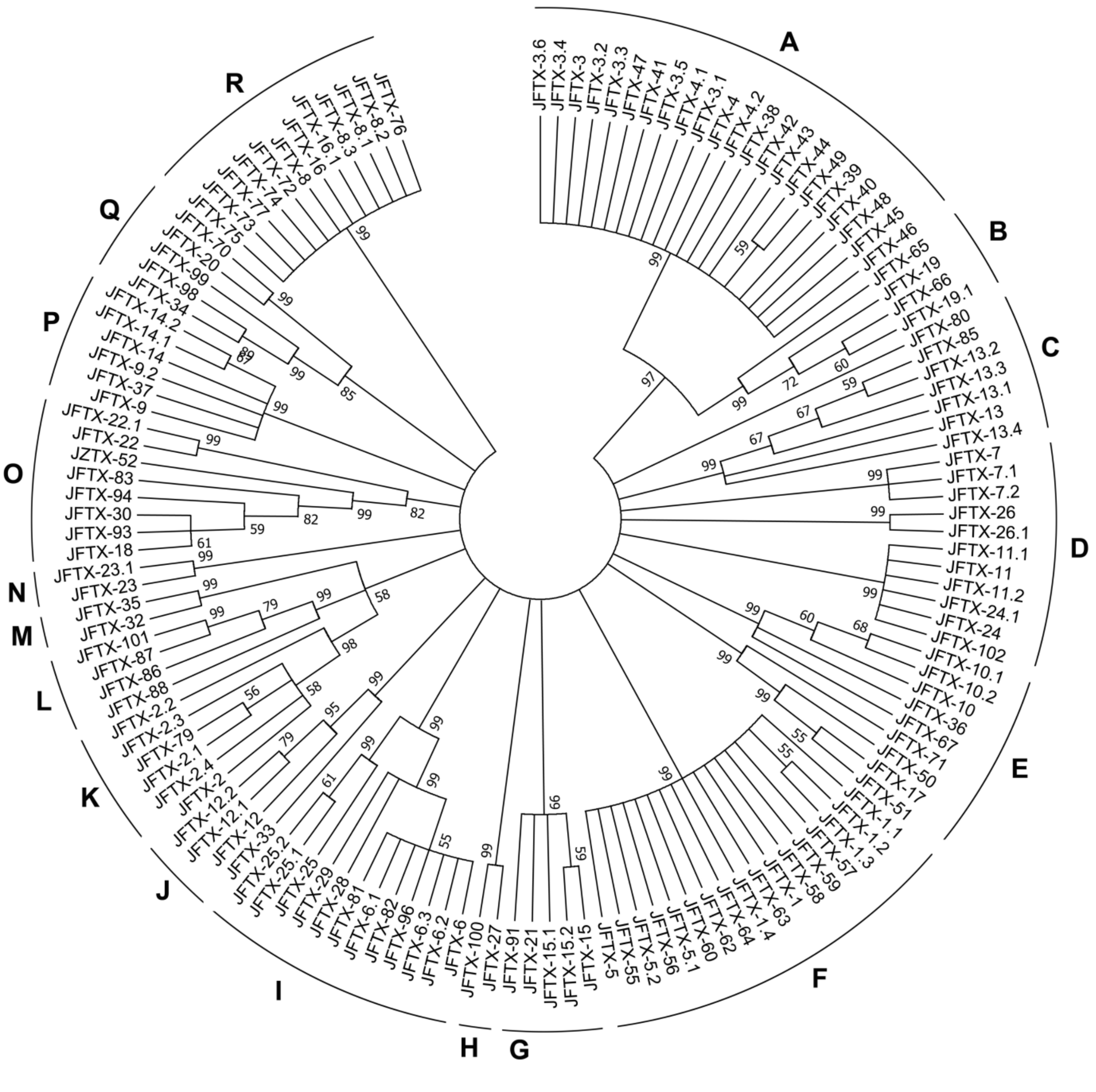

Figure 1. Phylogenetic tree of putative toxin precursors from *S. jiafu* venom glands. The phylogenetic analysis was conducted by using the neighbor-joining method of the MEGA 7 software package.
